# Affirm My Identity, Save My Life: A Scoping Review of Suicide Prevention Efforts for LGBTQ + Youth in K-12 Schools

**DOI:** 10.1007/s12310-025-09797-4

**Published:** 2025-08-09

**Authors:** Sarah M. Stilwell, Briana A. Scott, Heather Murphy, Esther Lee

**Affiliations:** 1 Department of Health Behavior and Health Equity, University of Michigan School of Public Health, University of Michigan, Ann Arbor, MI, USA; 2 Institute for Firearm Injury Prevention, University of Michigan Office for Vice President of Research, Ann Arbor, MI, USA

**Keywords:** LGBTQ youth, Suicide prevention, Suicidality, School-based programs, Scoping review

## Abstract

Suicidality among adolescents is a significant public health issue, with suicide being the second leading cause of death in the 10–14 year-old age group (CDC, 2024). Youth identifying as LGBTQ + (lesbian, gay, bisexual, transgender, and/or questioning/queer), in particular, experience disproportionately high rates of suicidal thoughts and behaviors (STB), often exacerbated by bullying, stigma, discrimination, and lack of support. Research indicates that protective factors, such as supportive adults, inclusive policies, and safe school environments, can reduce the risk of STB. Schools are uniquely positioned to implement suicide prevention strategies, including anti-bullying programs, LGBTQ + curricula, and mental health training for students and staff. This scoping review investigates the landscape of school-based efforts to prevent STB among LGBTQ + youth in K-12 schools over the past three decades (1994–2024). Focusing on implementation strategies, approaches, and design elements, this scoping review seeks to understand: What suicide prevention efforts have been implemented in K-12 schools to support LGBTQ + youth? Findings from the review suggest that school-based efforts (*N* = 5), such as inclusive anti-bullying policies, LGBTQ + curricula, and support networks like gender-sexuality alliances (GSAs), can reduce suicidal thoughts and behaviors among LGBTQ + youth. However, gaps remain in understanding the long-term effectiveness of these efforts, highlighting the need for more comprehensive, evidence-based strategies that address both individual and community-level factors.

## Introduction

The prevalence of suicide among adolescents has escalated in recent years, emerging as a critical public health concern (Glenn et al., 2020). Suicide is the second leading cause of death among adolescents in the 10–14 age group, and the third among those in the 14–18 age group in the USA (Centers for Disease Control & Prevention, 2023; Ivey-Stephenson, 2020). In 2021, suicide accounted for 18.6% of deaths among adolescents in the 14–18 age group (Centers for Disease Control & Prevention, 2023). While suicide deaths represent a significant portion of suicidality (encompassing suicide death, nonfatal suicide ideation, plan attempts, and injury due to attempts) among adolescents, the rates of suicidal thoughts and attempts have remained high and have consistently increased from 2009 to 2021 (Centers for Disease Control & Prevention, 2020).

Despite high rates of suicidal thoughts and behaviors (STB) among all adolescents, LGBTQ + youth (lesbian, gay, bisexual, transgender, and/or questioning/queer) experience disproportionately high rates (Gorse, 2022; Ream, 2022; Wang et al., 2021). LGBTQ + youth are five to eight times more likely to attempt or contemplate suicide compared to their heterosexual and cisgender peers (Almeida et al. 2009; Taliaferro & Muehlenkamp, 2017; Toomey et al., 2018). Further, myriad correlates may exacerbate STB in LGBTQ + youth, including, but not limited to, victimization, stigma, discrimination, bullying, sexual abuse, and lack of social support (Hatchel et al., 2021; Wang et al., 2023).

The increased risk of STB among LGBTQ + youth is often linked to adverse mental health outcomes, like depression and anxiety, which are known to contribute to these thoughts and behaviors (King, 2001). Additionally, LGBTQ + adolescents may face rejection from family members and peers, further exacerbating their vulnerability to mental health issues and STB (Madireddy & Madireddy, 2022). Addressing the rising rates of adolescent STB, particularly among LGBTQ + youth, requires comprehensive strategies that include mental health education, early intervention, and the promotion of supportive environments (Green et al., 2022a).

### Challenges and Risk Factors Contributing to STB in LGBTQ + Youth

The lived experience of LGBTQ + youth is often characterized by unique challenges and adversities that increase their risk of STB (Haas et al., 2010; Hatchel et al., 2019; Marraccini et al., 2022). These challenges often stem from discrimination, rejection, and societal stigma, which can lead to feelings of isolation and marginalization (Hatchel et al., 2019; Marraccini et al., 2022). Research demonstrates that across the lifespan, LGBTQ + individuals frequently face discrimination in the form of personal rejection, hostility, and social exclusion (Hatchel et al., 2019; Marraccini et al., 2022). A major contributing factor to the heightened suicide risk among LGBTQ + youth is cumulative minority stress, which reflects the ongoing psychological strain caused by these negative experiences (Green et al., 2022b). While these risk factors are recognized, there is still limited understanding of how experiences such as stress, marginalization, structural violence, and discrimination based on sexual orientation or gender identity are linked to suicidal behaviors (Hass et al., 2010)

Perceived stigma is a key factor contributing to the suicide risk of LGBTQ + youth. Discrimination, bullying, and historical oppression of sexual minorities can intensify feelings of alienation, anxiety, and depression (Aranmolate et al., 2017). Research demonstrates that social connectedness, particularly within supportive peer groups, can act as a protective factor by buffering the harmful effects of stigma (Kaniuka et al., 2019). However, many LGBTQ + youth also face identity-specific stressors, including family rejection, bullying, and harassment, which not only contribute to emotional distress but also increase potential risk of STB (Aranmolate et al., 2017; Liu & Mustanski, 2012).

Researchers have demonstrated that elevated suicide risk among LGBTQ + youth has been linked to factors such as lack of supportive environments and experiences of victimization (Hatchel et al., 2021; Madireddy & Madireddy, 2022; Marraccini et al., 2022). Family support and acceptance are critical in mitigating the likelihood of suicide attempts (Madireddy & Madireddy, 2022). Additionally, programs designed to reduce bullying, improve peer relationships, and offer mental health support can reduce the negative impacts of stigma (Fish, 2020; Goldbach et al., 2021). Schools also play an essential role by fostering inclusivity, providing safe spaces, and enacting anti-discrimination policies (Leung et al., 2022; Russell et al., 2021). By implementing these strategies and promoting greater social acceptance, we can help create safer, more affirming spaces for LGBTQ + youth, ultimately reducing the risk of suicide and improving mental health outcomes for this vulnerable group.

### The Role of Schools as a Protective Factor

Schools play a critical role in preventing suicidal thoughts and behaviors (STB) and providing essential resources to youth, including access to mental health care (Ayer & Colpe, 2023; Hatchel et al., 2021; Kalafat, 2003; Whitaker et al., 2016) Given that adolescents spend a significant portion of their time in school, these institutions are uniquely positioned as key agents of socialization, shaping students’ identities and providing a vital context for support (Arnett, 2013). Some studies have identified an association between supportive school environments, including trusted adult relationships, anti-bullying policies, and safe spaces, and reduced risk of suicidal thoughts and behaviors among LGBTQ + youth (Hatchel et al., 2019; Rivas-Koehl et al., 2022; Wang et al., 2023). Additionally, factors such as the presence of Gender-Sexuality Alliances (GSAs), LGBTQ + inclusive health programs, professional development on inclusive education practices, and the integration of LGBTQ + relevant content into the curriculum can further protect against STB and enhance the well-being of LGBTQ + students (Marraccini et al., 2022; Russell & Fish, 2016).

Schools that promote positive mental health resources and education on topics disproportionately affecting LGBTQ + youth, such as STB, are essential in reducing the stigma and discrimination these students often face (Johns et al., 2019; McDermott et al., 2023). By providing education on recognizing the signs and symptoms of STB and offering support services, schools can help mitigate the harmful effects of isolation, harassment, and marginalization, which are risk factors for suicide among LGBTQ + youth. These protective factors contribute to fostering resilience and support, helping LGBTQ + students navigate developmental milestones and encouraging overall positive youth development. In this context, school environments that prioritize inclusivity and mental health resources are pivotal in preventing STB and promoting the well-being of LGBTQ + youth.

School-based STB prevention efforts have been categorized into several approaches: 1) universal curricular education, 2) gatekeeper training, 3) school-wide suicide screening programs, and 4) enhancing individual protective factors (Granello & Zyromski, 2018). Universal curricular education involves classroom-wide programs designed to raise awareness about STB and coping skills programs (e.g., Youth Aware of Mental Health; Lindow et al., 2019), including recognizing signs and symptoms of distress among peers (Katz et al., 2013). Additional examples of universal curricular education might include peer-led interventions (e.g., Sources of Strength; Wyman et al., 2025) and elementary school classroom interventions (e.g., Good Behavior Game; Flower et al., 2014). Gatekeeper training equips staff, students, and other school personnel with the skills to identify at-risk students and respond appropriately by connecting them to mental health resources (Singer et al., 2019). School-wide suicide screening programs help identify at-risk youth through regular assessments, ensuring early intervention and support (Granello & Zyromski, 2018). Enhancing protective factors, such as parent education, also plays an essential role in reinforcing the support network for LGBTQ + youth (Granello & Zyromski, 2018). While we adopt Granello and Zyromski’s (2019) use of “protective factors” to describe protective school-based STB strategies, it is important to note that this framing may not reflect the protective factors that are empirically demonstrated to buffer and moderate the effects of specific risks, i.e., stress-buffering (Masten et al., 2008, 2022). For consistency, we retain the terminology posited by Granello and Zyromski (2019), but recognize the conceptual ambiguity highlighted by Masten and colleagues ().

While schools are uniquely positioned to support LGBTQ + youth and prevent STB, critical gaps remain in the research on specific interventions, programs, and policies implemented in K-12 schools. Existing literature lacks a comprehensive understanding of how school-based efforts address the needs of LGBTQ + students, particularly in STB prevention. This research seeks to fill this gap through a scoping review, systematically identifying and evaluating the interventions, programs, and policies across schools. By understanding these efforts, the study will provide insights into how schools can better mitigate STB and emphasize the need for targeted, evidence-based support systems for LGBTQ + youth.

## Method

### Search Strategy

The primary objective of this scoping review was to identify efforts that have been implemented within K-12 schools to prevent STB among LGBTQ + youth. We reviewed published literature to identify efforts to reduce or mitigate STB in K-12 schools. We used a protocol informed by the Preferred Reporting Items for Systematic Reviews and Meta-Analysis (PRISMA) guidelines to search bibliographic databases, screen articles, apply inclusion and exclusion criteria, and select relevant literature for this scoping review (Moher et al., 2009; Tricco et al., 2018). The completed PRISMA-ScR checklist is shown in Appendix A (Tricco et al., 2018). With the help of a trained librarian, we developed a comprehensive search to find publications using the following databases: PubMed, ERIC, Education Abstracts, Social Science Abstracts, LGBTQ + Source, and Scopus. An example of search terms used for one of the databases is included in Appendix B.

We restricted our search to English-only articles and collected all database results published in the past 30 years (1994–2024). We searched for *suicide* and *K-12* and *LGBTQ* + . Subsequently, the text words in the titles and abstracts of relevant articles and the index terms used to describe the articles were used to develop criteria for our final review sample.

### Population of Interest

#### K-12 LGBTQ + Youth

For the current review, the population of interest was considered youth within K-12 schools who identify as part of the LGBTQ + population (lesbian, gay, bisexual, transgender, and/or questioning/queer). Included studies must explicitly describe implications for LGBTQ + students (e.g., articles that only describe diversity, equity, and inclusion, or cultu ally-sensitive training without describing implications for LGBTQ + students were not included).

### Concept

#### STB Prevention Efforts

We operationalized efforts to prevent STB as a program, policy, professional development, training, or intervention implemented within the school setting, including before, during, and after school on school grounds. These are programs that seek to prevent or reduce behaviors related to deliberate ending or attempting to end one’s life or experiencing suicidal ideation (Nock et al., 2008; Sufrate-Sorzano et al.; 2023). These efforts could be intended for audiences including students, teachers, administrators, and school community members or policies designed for implementation at the school, district, or state level. Efforts that were included in this review specifically sought to prevent STB or reduce ideation in LGBTQ + youth.

### Inclusion and Exclusion Criteria

The included articles were English-language articles published in peer-reviewed journals that reported on K-12 school-based efforts to reduce STB for LGBTQ + youth. We included efforts that took place before, after, or during school activities and were based in the United States (international efforts were not included in this review). Gray literature (e.g., reviews, editorials, dissertations, conference abstracts, and commentaries) was excluded from this review.

### Study Selection

**Figure 1:**
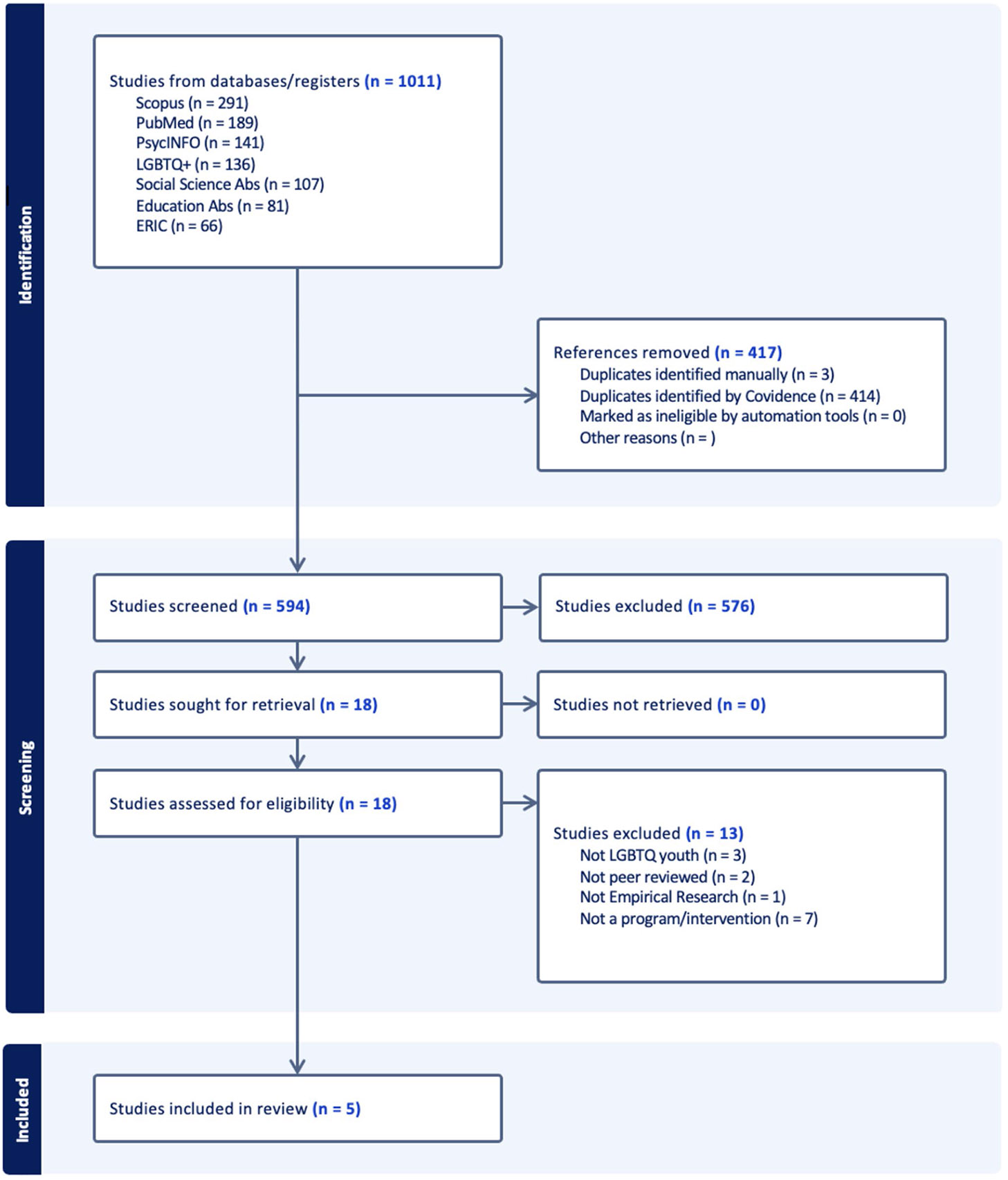
PRISMA flow diagram

Using Covidence (a web-based review software), we conducted a title and abstract review to determine if we should include the paper in our review. For those included, we performed a full-text review for each article. Two reviewers blindly and independently screened each title and abstract for inclusion/exclusion criteria. The full study team met to resolve discrepancies. The team followed a similar procedure for the full-text review, whereby two reviewers blindly and independently reviewed the full text of each article that passed title/abstract screening, and the study team met to resolve disagreements. After the full-text review was completed, reviewers extracted data from the full text of articles that met the inclusion criteria. All excluded studies were documented with the reasons for their exclusion through a flow chart and table in adherence to the PRISMA flowchart (Fig. 1).

### Data Extraction

The same study team that conducted the screening extracted data for each included study in the review. The structure of the data extraction was discussed with the study team, and the final extraction included the following study elements: author/publication year, variables of interest, description of effort to prevent STB, study setting, LGBTQ + sample (as characterized by study authors), results, and directions for future research. Study characteristics are also reported in a simplified format in Table 1.

## Results

After removing 417 duplicate articles, we screened 594 articles, of which 576 were excluded on the grounds that they did not meet our inclusion criteria. Full-text articles (*N* = 18) were assessed for eligibility and excluded based on our criteria. Of those, seven were excluded because they did not report on STB prevention efforts, did not include LGBTQ + youth, were not peer-reviewed, or were not considered empirical research. A final sample of five articles was included in the review. All five articles employed observational research designs; none of the studies identified used causal designs. Thus, only associations can be drawn from the results of the articles included below, rather than causal inference.

The following section presents results, organized by individual study, and using the author’s terminology and variable operationalizations. The studies are presented by unit of implementation, from most narrow (school health clinic) to broadest implementation setting (statewide/multi-state).

### Study 1: Zhang et al. (2020)

#### Authors, Variables, and Study Overview:

Zhang et al. (2020) examined the association between school-based health centers and the mental health of Sexual Minority Youth (SMY). The outcome variables included were suicidal ideation and suicide attempts within the past 12 months. An additional outcome of depressive episodes within the past 12 months was included.

#### Description of effort:

The authors analyzed data from the 2015 Oregon Healthy Teens Survey (OHT) to determine if there was an association between the presence of school-based health centers and three mental health indicators (depressive episodes, suicidal ideation, and suicide attempts in the past 12 months. School-based health centers provide health services for enrolled students on campus, removing many barriers to youth healthcare access (e.g., transportation). Further, sexual minority youth reported higher levels of mental health concerns and utilized health services more than their heterosexual peers.

#### Study Setting, Duration, and Sample Characteristics:

The survey sample included 13,608 students across 137 Oregon public middle and high schools; 50.2% identified as female, 89.3% identified as White, and 23.6% identified as Hispanic. The average age of students was 16.6 years. Of the 137 schools, 26 had School-Based Health Centers (SBHCs).

#### LGBTQ + Sample:

Of the total sample of 13,488 students, 1,501 (11.3%) identified with a minority sexual orientation. Broken down further, 199 (1.4%) youth identified themselves as Lesbian or Gay, 700 (5.3%) as bisexual, 267 (1.8%) as something else, and 335 (2.9%) as do not know/not sure.

#### Results:

SMY at schools with a SBHC had a relative reduction in likelihood for STB in the past 12 months compared to SMY at schools without a SBHC. This suggests that SBHCs could be a key component of mental health prevention and intervention for SMY.

### Study 2: Hatzenbuehler & Keyes (2013)

#### Authors, Variables, and Study Overview:

Hatzenbuehler and Keyes (2013) evaluated the degree to which statewide school anti-bullying policies were inclusive of sexual orientation and were also associated with reduced prevalence of student STB among lesbian and gay youth. The outcome variable included in this study was self-reported suicide attempts in the past 12 months. Peer victimization was included as a covariate to adjust for the association between LGBTQ + identity and STB.

#### Description of effort:

Using data from the Oregon Department of Education, the authors examined school anti-bullying policies at the district level. They determined how inclusive the policies were (least, medium, and most) for lesbian and gay youth. Policy data were compared with associations of student self-reported suicidality using data from the Oregon Health Teens (OHT) survey.

#### Study Setting, Duration, and Sample Characteristics:

This study included an overall sample of 31,852 11th-grade public school students from 34 counties in Oregon. The students completed the OHT survey between 2006 and 2008.

#### LGBTQ + Sample:

The LGBTQ + sample in this study was based on student self-report, and included 301 (.9%) students who identified as gay or lesbian, and 1,112 students (3.3%) who identified as bisexual.

#### Results:

Lesbian and gay youth in counties with fewer school districts that had inclusive anti-bullying policies were, on average, 2.25 times the estimated odds of attempting suicide in the past year compared to those in counties with more inclusive policies. Inclusive policies were linked to a significantly lower risk of suicide attempts, even after accounting for factors including sex, race/ethnicity, and peer victimization. In contrast, anti-bullying policies that did not specifically include sexual orientation did not show any significant effect on reducing suicide attempts among LGBTQ + youth.

### Study 3: Krantz et al. (2024)

#### Authors, Variables, and Study Overview:

Krantz and colleagues (2024) evaluated the effect of statewide anti-bullying laws and local interventions on LQBTQ youth suicidality. LGBTQ youth in this study are youth who self-identify as a sexual orientation other than straight/heterosexual, as a gender identity other than cisgender, or both. For the STB outcome variable, participants self-reported whether or not they attempted suicide within the last 12 months. Additional outcomes in this study include perceptions of affirming school spaces and physical threats or harm.

#### Description of effort:

In collaboration with the Trevor Project, the authors examined predictors, including state-level anti-bullying laws and school-level strategies, such as the percentage of schools with a Gay-Straight Alliance (GSA) club and LGBTQ-specific sexual education, on STB among LGBTQ youth.

#### Study Setting, Duration, and Sample Characteristics:

The study included an overall sample of 27,697 LGBTQ youth (aged 13–24 years old) from 44 states in the U.S. The students completed the Trevor Project survey in 2021.

#### LGBTQ + Sample:

When asked about their sexual orientation, participants self-identified as lesbian (13%), gay (12%), bisexual (31%), pansexual (20%), queer (10%), asexual (9%), or questioning (4%). When asked about their gender identity, participants identified as cisgender (33%), transgender or non-binary (48%), or questioning (19%).

#### Results:

The presence of statewide anti-bullying laws, specifically those with protections for LQBTQ youth, was associated with a lower risk of suicide attempts among LGBTQ youth. However, universal strategies and policies not specific to the LGBTQ population did not reduce the risk of suicide attempts among LGBTQ youth.

### Study 4: Kuhlemeier (2024)

#### Authors, Variables, and Study Overview:

This study sought to examine the impact of state school policies and practices designed to reduce disparities experienced by sexually and gender-diverse students on STB. The outcome variable included in this study was self-reported suicidal thoughts and one or more suicide attempts in the past 12 months. No additional outcomes related to prevention of STB were included in the study.

#### Description of effort:

Kuhlemeier (2024) utilized data from the 2017 and 2019 New Mexico Youth Risk and Resiliency Survey and School Health Profiles to examine the impact of four strategies recommended by the Centers for Disease Control and Prevention (CDC) —school-sponsored GSAs, safe zones/spaces, Sexually and Gender-Diverse (SGD)-specific anti-bullying policies, and professional development for staff on issues affecting SGD youth on suicidality among lesbian, gay, bisexual, questioning, and transgender/gender-diverse students.

#### Study Setting, Duration, and Sample Characteristics:

Individual-level data came from the 2017 and 2019 New Mexico Youth Risk and Resiliency Survey (part of the Youth Risk Behavior Surveillance System) and the 2016 CDC School Health Profiles. The School Health Profiles surveys were administered to principals and health educators to assess school policies and practices. Principals from 90 schools in New Mexico responded to the survey, representing more than half of the total number of schools that participated in those years. Only students who attended a sampled school (*N* = 165) were included in the analysis, resulting in a final sample of 22,993 students.

#### LGBTQ + Sample:

The average age of the participants was 15.7 years. Approximately 2.7% identified as transgender or gender diverse, 3.3% as gay or lesbian, 9.7% as bisexual, and 4.6% as questioning their sexuality. Nearly half (48.1%) of the sample identified as Hispanic or Latinx, while 20% were non-Hispanic White, 21.8% American Indian/Alaska Native, 7.9% Black or African American, and 2.1% Asian or Pacific Islander.

#### Results:

Attending a school with a gender and sexualities alliance was linked to lower odds of STB among questioning youth compared to other heterosexual and SGD youth. There was no significant relationship between positive school climate strategies and SGD youth’s STB.

### Study 5: Seelman and Walker (2018)

#### Authors, Variables, and Study Overview:

Seelman and Walker (2018) explored the effect of general and enumerated multi-state anti-bullying laws on STB among lesbian, gay, bisexual, and questioning (LGBQ) high school students. For the suicide outcome variable, participants self-reported if they ever seriously considered attempting suicide in the last 12 months or the number of suicide attempts over the previous 12 months. Additional outcomes included bullying victimization, fear-based absenteeism, in-school threats, or injury with a weapon.

#### Description of effort:

Youth Risk Behavior Survey (YRBS) data across 22 states between 2005 and 2015 were compared with the presence of general and enumerated anti-bullying laws that include sexual orientation as a protected class to explore outcomes for LGBQ youth.

#### Study Setting, Duration, and Sample Characteristics:

The authors utilized a quasi-experimental design to analyze YRBS data and data regarding the presence of anti-bullying laws. The sample population for this study included 286,568 total possible cases of youth who completed the YRBS survey and were asked about their sexual identity.

#### LGBTQ + Sample:

A weighted estimate of 10.5% of high school students surveyed reported a sexual identity of lesbian, gay, bisexual, or not sure.

#### Results:

The presence of general or enumerated anti-bullying laws did not have a significant impact on suicide ideation or attempts for LGBQ youth. One model for suicide attempts found enumerated anti-bullying laws reduced suicide attempts by 3.3% for LGBQ youth in a given year; however, this was not robust to the exclusion of youth that reported “not sure” for their sexual orientation, and was not statistically significant when examined by sex and age.

## Discussion

This review highlights several critical gaps in the literature regarding STB prevention efforts for LGBTQ + youth, and the insights underscore both challenges and opportunities for future research. The most crucial takeaway from this review is the dearth of empirically published studies examining K-12 school-related interventions specifically developed for preventing suicide and reducing STB in LGBTQ + youth. Most existing research efforts focus on experiments that examine policies already in place (e.g., Hatzenbuehler & Keyes, 2013), limiting the conclusions that can be drawn from such studies (e.g., associations rather than causal inferences). However, studies included in this review consistently demonstrate that inclusive and supportive LGBTQ + policies, such as anti-bullying protections and inclusive curricula, are associated with lower odds of STB among LGBTQ + youth. This is an important and encouraging finding, suggesting that schools can play a unique role in implementing systemic and policy-level changes that can have meaningful protective effects against suicidal thoughts and behaviors among LGBTQ + students. While such studies are valuable in understanding the context and current practices, they do not provide the rigorous, experimental evidence needed to evaluate the true effectiveness of school interventions. The absence of Randomized Controlled Trials (RCTs) or other robust experimental designs that evaluate the direct impact of universal programs, such as Signs of Suicide, that seek to reduce suicidality among LGBTQ + students is a critical gap in the field. This is a crucial limitation, as it prevents the development and replication of evidence-based guidelines for schools. For example, there may be common school practices of prevention programs (e.g., risk assessments and gatekeeper training) that have differential effects for LGBTQ + students depending on how inclusive or affirming the implementation is. Given the high level of stigma experienced by this population, effect sizes reported in research may be confounded by factors such as reduced engagement, perceived safety, or cultural relevance. Thus, while the existing evidence provides an important foundation, future research should prioritize the development, testing, and eventually replication of tailored interventions that seek to prevent suicide specifically for LGBTQ + students, particularly through RCTs or other rigorous evaluations.

Across the studies included in this review, there was considerable variability in how LGBTQ + populations were defined, measured, and included, particularly regarding the distinction between gender identity and sexual orientation. This inconsistency complicates efforts to draw generalized conclusions about the most effective approaches to LGBTQ + suicide prevention in schools. Furthermore, only two studies (Krantz et al., 2024 and Kuhlemeier, 2024) explicitly include transgender youth, a group that faces unique challenges and risks. This elucidates another critical gap in the literature, with a clear need for more research that specifically addresses the experiences of transgender and non-binary youth (Greytak et al., 2013). Future studies should seek to standardize the measurement of gender identity and sexual orientation and explore the intersectionality of these identities with other factors such as race, socioeconomic status, and mental health, as research has shown that LGBTQ + youth from marginalized groups may face higher risks of suicidality (Madireddy & Madireddy, 2022; Williams et al., 2019).

Additionally, studies in this review generally focused on older adolescent populations, as all five examples include students in middle or high school. This reveals a potential research gap, overlooking younger students who might benefit from earlier interventions. Early adolescence, or even earlier stages of development, could be critical windows for intervention, as LGBTQ + youth often experience bullying, discrimination, and mental health challenges beginning in elementary school (Gower et al., 2018). Embedding LGBTQ + -affirmative practices, such as inclusive curricula, supportive teacher training, and anti-bullying policies, in the early years of K-12 education could offer a proactive approach to addressing these challenges before they escalate into more severe mental health issues, including STB (Davis et al., 2024). Intervening earlier could help foster a more supportive environment, reduce feelings of isolation, and promote resilience, all of which are essential for preventing STB (Ungar et al., 2014). Furthermore, by focusing on younger students, schools could normalize LGBTQ + identities and create safe spaces for all students, regardless of their sexual orientation or gender identity, thereby building long-term protective factors (Sadowski, 2017). Future researchers may consider examining how early interventions in K-12 education impact the mental health and well-being of LGBTQ + youth and explore the potential benefits of fostering an inclusive and affirming school environment from the earliest stages of education.

Intersectionality is another important consideration for future research. This review underscores the need to explore how multiple identities (e.g., race, gender, and sexual orientation) may intersect to influence STB among LGBTQ + youth, and the role that cumulative minority stress might play (Green et al., 2022b). Research has demonstrated that LGBTQ + youth who belong to multiple historically marginalized groups may face compounded stressors, leading to higher rates of STB (Gomez et al., 2011; Green et al., 2022b). Therefore, future studies should examine how school-based interventions can be tailored to address the unique experiences of LGBTQ + youth who may face intersecting forms of discrimination. For example, researchers might consider including a more diverse sample to understand how identities such as race/ethnicity, religion, or disability status may affect results (Zhang et al., 2020).

In terms of intervention strategies, this review highlights the importance of both universal (e.g., school-wide anti-bullying policies) and specific preventative interventions for LGBTQ + youth (e.g., enhancing individual protective factors and tailored interventions). Universal interventions are associated with youth feeling safer and experiencing reduced suicidality, and allow schools to support LGBTQ + youth without requiring them to disclose their identity, thus avoiding additional stressors that may arise from coming out in school settings (Granello & Zyromski, 2018; Katz et al., 2013). There are varying levels of policies that may affect school systems, including at the school and state levels that contribute to school-wide policies and procedures. These may consist of suicide prevention policies that specify qualifications for screening and referral processes for concerns (Singer et al., 2019) and state policies related to school staff suicide prevention training (Kreuze et al., 2017). Specific interventions tailored to LGBTQ + youth are also important. For example, studies such as Goldbach et al. (2018) demonstrate that LGBTQ + youth are more likely to engage with LGBTQ-specific crisis hotlines because these services are perceived as identity-affirming. These findings suggest that interventions that explicitly recognize and validate LGBTQ + identities are crucial to ensuring that youth feel supported and understood. Future research should seek to proactively incorporate LGBTQ + youth voice in the development and implementation of interventions to prevent STB (Wagaman, 2015). Furthermore, future efforts should seek to explore not only explicitly labeled LGBTQ + programs but also other forms of support that may be indirectly helping to reduce suicidality among LGBTQ + youth. This could include examining broader protective factors, such as positive school climate, teacher-student relationships, and peer support programs, to gain a more comprehensive understanding of what contributes to the mental health and well-being of LGBTQ + students. By exploring the effects of a combination of universal and specific interventions to support LGBTQ + youth, and leveraging youth voice, long-term future efforts can better support all youth and community members.

This review emphasizes the need for more targeted research into school-based suicide prevention for LGBTQ + youth, with a focus on developing and testing interventions that are both effective and inclusive of the diverse experiences within the LGBTQ + community. Longitudinal research could be used to assess the effects of policies and legislation, and examine the fidelity of policy implementation to determine how closely schools implement policies outlined in school guidelines, while leveraging additional qualitative and quantitative methodological approaches can help researchers better understand the effects of inclusive school policies and practices on LGBTQ + youth mental health (Hatzenbuehler & Keyes, 2012; Krantz et al., 2024; Kuhlemeier, 2024). Addressing gaps, such as the inclusion of transgender youth, younger populations, and intersectional identities, is crucial for developing evidence-based guidelines that can inform school policies and practices across the nation.

### Limitations

Although we did not identify any published studies that employed an experimental design to test an intervention to reduce STB among LGBTQ + youth as part of this review, we did not include a gray literature search in our methodological approach. As a result, unexamined STB prevention efforts may be implemented in schools that are not documented in academic literature, such as informal programs, internal unpublished evaluations, teacher training, or community collaborations. These interventions may not have undergone formal evaluation or testing but could still offer valuable insights into effective strategies. Future researchers may include gray literature, including unpublished reports and program evaluations, to uncover additional interventions and assess their impact, ultimately contributing to a more comprehensive understanding of effective STB prevention efforts for LGBTQ + youth.

Another challenge is adequately powering analyses, particularly when focusing on subpopulations such as LGBTQ + youth. This issue may arise due to factors such as low outcome base rates, funding limitations, the need for parental consent, reluctance among some youth to disclose their sexual identity, and the inherent challenge of distinguishing between elevated suicide attempt risk and actual suicide mortality risk. Moreover, studies included in this review do not account for youth who have died from suicide. As a result, findings may be subject to selection bias, potentially underestimating the true burden or prevalence of STB within this population, and should be interpreted within the context of this limitation.

In addition, interventions included in this review demonstrated inconsistent presentation of findings, which potentially hinders the integration and comparison of results across studies. The breadth and heterogeneity of the included studies pose challenges for interpretations and drawing conclusions. Given the variation in study design, populations, and focal area, there are limitations in ability to uniformly synthesize results or make broad generalizations. Thus, further research is recommended to better understand underlying factors contributing to these inconsistencies and to develop standardized approaches that enhance comparability and generalizability of future findings.

Finally, inclusion for the current review did not account for suicide prevention efforts outside the school setting, such as community-based programs (Allen et al., 2012), online support (Banyard et al., 2022), or services offered through organizations like spectrum centers or mental health clinics (Zullo et al., 2021). As students may seek help from these external resources, understanding their impact is crucial for developing a comprehensive understanding of the support networks available to LGBTQ + youth. STB prevention and postvention efforts should not only address school environments but also integrate community-based resources, family involvement, and other protective factors that can support LGBTQ + youth and buffer against both the risk of suicidal behavior and the effects of suicide within their communities. While the current scoping review followed PRISMA-ScR guidelines, and included the respective flow chart and checklist, the research protocol was not registered. As such, future research efforts, including those that examine prevention efforts at an ecological level, can adapt the current protocol, register the modified version, and help contribute to a larger evidence-based understanding of how school-based efforts fit into the broader landscape of LGBTQ + youth mental health to improve outcomes for all.

## Conclusion

While this review provides valuable insights into the need for more evidence-based efforts that promote STB prevention for LGBTQ + youth, its limitations underscore the need for future research that expands the scope of inquiry. Incorporating gray literature and examining community-based interventions are essential for developing a more complete understanding of how to best support LGBTQ + youth in schools and beyond. Taking into account the broader support system for LGBTQ + youth and addressing methodological inconsistencies will contribute to creating a more nuanced and comprehensive approach to suicide prevention for this at-risk population, and communities at large.

## Supplementary Material

Supplementary Material

**Supplementary Information** The online version contains supplementary material available at https://doi.org/10.1007/s12310-025-09797-4.

## Figures and Tables

**Table 1:** Study and intervention characteristics, variables, and descriptions (*N* = 5)

Author (Year)	Study Setting & Description	LGBTQ+ Variable Construct	Suicide Variable Construct	Outcome
Hatzenbuehler & Keyes (2012)	Association between inclusiveness of 34 Oregon school district anti-bullying policies and STB among LGBTQ+ youth	Sexual identity (lesbian, gay, bisexual)	Self-reported suicidal thoughts and/or attempts in the past 12 months	Anti-bullying policies without specific protections for sexual orientation did not reduce LGBTQ+ youth suicide attempts. LGBTQ+ youth in counties with fewer district-level inclusive anti-bullying policies more likely to attempt suicide compared to those in counties with more inclusive policies.
Krantz et al. (2024)	Association between seven state and school-level prevention efforts and STB among LGBTQ+ youth in 44 states	Sexual orientation (lesbian, gay, bisexual, pansexual, queer, questioning, asexual) Gender identity (questioning, transgender or nonbinary)	Self-reported suicide attempts within the last 12 months	Statewide anti-bullying laws with specific protections for sexual orientation and/or gender identity were associated with a lower risk of suicide attempts among LGBTQ+ youth. Strategies and policies not specific to the LGBTQ+ population were not associated with a lower risk of suicide attempts among LGBTQ+ youth.
Kuhlemeier (2024)	Association between four CDC-recommended prevention strategies and STB among LGBTQ+ youth in 90 New Mexico high schools	Sexual identity (lesbian/gay, bisexual, questioning) Gender identity (transgender/gender diverse)	Self-reported suicidal thoughts and/or attempts in the past 12 months	Attending a school with a GSA was associated with lower odds of STB among questioning youth. Professional development for supporting LGBTQ+ youth, safe spaces with supportive staff, and inclusive anti-bullying policies were not associated with lower odds of STB among LGBTQ+ youth.
Seelman & Walker (2018)	Association between 22 general and enumerated state anti-bullying laws and STB among LGBTQ+ high school students	Sexual identity (gay/lesbian, bisexual, questioning/ not sure)	Self-reported suicidal thoughts and/or attempts in the past 12 months	General and enumerated anti-bullying laws were not associated with a reduced likelihood of STB among LGBQ youth.
Zhang et al. (2020)	Association between presence of school-based health center and STB among LGBTQ+ youth in 137 Oregon public middle and high schools	Sexual Minority Youth (lesbian/gay, bisexual, something else, don’t know/not sure)	Self-reported suicidal thoughts and/or attempts in the past 12 months	SMY at schools with school-based health centers had a relative reduction in the likelihood of suicidality in the past 12 months compared to SMY at schools without school-based health centers.

STB = Suicidal Thoughts and Behaviors, SMY = Sexual Minority Youth

## Appendix

### Search Terms for PubMed database search

1. ("Suicide"[Mesh] OR Suicid*[tiab]) AND ("Preventive Health Services"[Mesh] OR "Preventive Psychiatry"[Mesh] OR "Primary Prevention"[Mesh] OR "prevention and control" [Subheading] OR prophylaxis[tiab] OR prevent*[tiab])
2. "School Health Services"[Mesh] OR "Students"[Mesh:NoExp] OR "Schools"[Mesh:NoExp] OR "10th grade"[tiab] OR "11th grade"[tiab] OR "12th grade"[tiab] OR "1st grade"[tiab] OR "2nd grade"[tiab] OR "3rd grade"[tiab] OR "4th grade"[tiab] OR "5th grade"[tiab] OR "6th grade"[tiab] OR "7th grade"[tiab] OR "8th grade"[tiab] OR "9th grade"[tiab] OR "educational setting*"[tiab] OR "junior high"[tiab] OR classroom*[tiab] OR classroom*[tiab] OR highschool*[tiab] OR k-12[tiab] OR kindergarten*[tiab] OR school*[tiab] OR student*[tiab] OR teacher*[tiab]
3. "Bisexuality"[Mesh] OR "Health Services for Transgender Persons"[Mesh] OR "Homosexuality"[Mesh] OR "Sexual and Gender Minorities"[Mesh] OR "Transgender Persons"[Mesh] OR "2 spirit"[tiab] OR "bigender"[tiab] OR "bi sexual*"[tiab] OR "cross gender*"[tiab] OR "flexible gender"[tiab] OR "gender affirm*"[tiab] OR "gender atypical"[tiab] OR "gender binar*"[tiab] OR "gender confirm*"[tiab] OR "gender confusion"[tiab] OR "gender divers*"[tiab] OR "gender expression"[tiab] OR "gender flex*"[tiab] OR "gender fluid*"[tiab] OR "gender free"[tiab] OR "gender identi*"[tiab] OR "gender minorit*"[tiab] OR "gender non-binar*"[tiab] OR "gender non-conform*"[tiab] OR "gender non-binar*"[tiab] OR "gender non-conform*"[tiab] OR "gender questioning"[tiab] OR "gender self concept"[tiab] OR "gender varian*"[tiab] OR "male female binar*"[tiab] OR "male/female binary"[tiab] OR "men having sex with men"[tiab] OR "men who have sex with men"[tiab] OR "non-cisgender"[tiab] OR "non-normative gender"[tiab] OR "same-gender-lov*"[tiab] OR "sexual minorit*" [tiab] OR "sexual orientation"[tiab] OR "third gender*"[tiab] OR "trans adolescen*"[tiab] OR "trans child*"[tiab] OR "trans communit*"[tiab] OR "trans female*"[tiab] OR "trans feminine"[tiab] OR "trans identit*"[tiab] OR "trans individual*"[tiab] OR "trans male*"[tiab] OR "trans masculine"[tiab] OR "trans people*"[tiab] OR "trans person*"[tiab] OR "trans population*"[tiab] OR "trans sexual"[tiab] OR "trans specific"[tiab] OR "trans teen*"[tiab] OR "trans youth"[tiab] OR "two spirit"[tiab] OR "women loving women"[tiab] OR "women who have sex with women"[tiab] OR agender[tiab] OR asexual*[tiab] OR bigender[tiab] OR bigender[tiab] OR bisexual*[tiab] OR gay[tiab] OR gblt*[tiab] OR genderfluid[tiab] OR genderfree[tiab] OR genderqueer[tiab] OR glbt[tiab] OR glbtq[tiab] OR homosexual*[tiab] OR intersex*[tiab] OR lbgt*[tiab] OR lesbian*[tiab] OR lesbigay[tiab] OR lgbt*[tiab] OR msm[tiab] OR multisexual*[tiab] OR non-binary[tiab] OR non-binary[tiab] OR pangender[tiab] OR pansexual*[tiab] OR plurisexual*[tiab] OR polygender[tiab] OR queer*[tiab] OR transexual*[tiab] OR transfemini*[tiab] OR transgender*[tiab] OR transmasculine[tiab] OR transpeople[tiab] OR transsexual*[tiab] OR wsw[tiab]
